# Theories, models and frameworks of school nursing - a scoping review

**DOI:** 10.1186/s12912-025-03730-5

**Published:** 2025-09-10

**Authors:** Jana Kaden, Birte Berger-Höger

**Affiliations:** Institute for Public Health and Nursing Research, Department Evaluation and Implementation Research in Nursing Science, University of Bremen, Grazer Straße 4, D- 28359 Bremen, Germany

**Keywords:** School nursing, School health, Model, Framework, Theory, Concept, Approach, Health, Education

## Abstract

**Background:**

School nursing is a complex clinical specialty practice that varies across different countries. Theories, models and frameworks can inform nursing practice. This scoping review aims to explore the conceptualisation and operationalisation of school nursing in theories, models and frameworks.

**Methods:**

A scoping review was conducted according to the JBI Manual for evidence synthesis. To identify existing theories, models, frameworks and concepts of school nursing, we searched in the databases Medline/PsycInfo, CINAHL and ERIC from earliest date until 08 March 2024. There was no limitation regarding the study type, school settings and countries. For data analysis, theory and concept analysis was employed, supplemented by content analysis with additional type building. School nurses’ tasks were clustered according to the WHO guideline on school health services.

**Results:**

We identified two theories, eleven different models, and seven different frameworks that were reported in 28 publications. Both identified theories aim to empower school nurses, and provide orientation. Among the identified models and frameworks, we identified three types, which were “Role and practice models/frameworks”, “Organisation and delivery models/frameworks” and “Qualification models/frameworks”. Definitions and aims of school nursing varied in the identified literature. Aims included improving or supporting student health, health care access, education and school attendance. Further frameworks aim to strengthen the role of a school nurses or to promote their professional development. Tasks of school nurses comprise health promotion, health education, clinical assessment and preventive interventions. Leadership, evaluation and coordination were identified as key elements of school nursing, associated with autonomy. Most of the theoretical foundations describe a Bachelor’s degree as minimum qualification for school nurses.

**Conclusion:**

The identified theoretical foundations are intended to support school nurses in developing their role, tasks and structure in relation to the targeted aims. School nurses’ complex role profiles require appropriate competencies that go beyond a qualification on a Bachelor’s degree level. The results could inform practice implementation, development and further research by reflecting and evaluating the existing practice. For countries without widespread implementation of school nursing, the identified theoretical foundations could serve as a base for further implementation steps.

**Supplementary Information:**

The online version contains supplementary material available at 10.1186/s12912-025-03730-5.

## Background

School nursing is a complex clinical speciality of nursing practice and part of school health [1–3]. The aims of school nursing include support of the well-being, academic success, lifelong achievement and health of students [4, 5]. School nurses interact in different settings with diverse persons, including the students, families, school staff and community residents [5, 6]. School nurses engage with students (and their families) both inside and outside the school setting and have a crucial role in identifying and addressing their (unmet) health needs. They offer health promotion over a long period of time and develop the school into a health-promoting environment [6–9].

Definitions, approaches, tasks and responsibilities related to school nursing vary across countries and states [10, 11]. For instance, whether public health nursing is part of school nursing [12], or school nursing is part of public health nursing [13, 14] depends on the national context, related approaches and understanding of public health nursing. However, school nursing has its roots in public health nursing [14, 15]. There is a strong connection between the nursing profession and public health. School nurses use not only public health nursing knowledge and skills [12] but also elements of primary health care, emergency care, paediatrics, mental health care, community care and social science [1, 13].

There is also a variability in the school nurses’ qualifications [13]. The role of the school nurse has changed over the last 30 years. Previously in 1990s, school nurses were expected to work with a strictly task-focused health promotion approach. The roles of school nurses have become more complex due to the increasing burden from chronic and non-communicable diseases, and subsequently increasing complexity of social and health care needs for children and adolescents in schools [7, 14, 16]. This broad spectrum of tasks in the constantly changing educational environment is realised differently [15]. As the only healthcare professionals in schools, school nurses often have the advantage of working autonomously. Despite the freedom and autonomy entailed within the jobs, school nurses, especially the new nurses, could face many difficulties associated with working in isolation [17]. School nurses build a bridge between the health and education sectors, requiring a close collaboration and coordination among staff in both fields. However, interdisciplinary collaboration in school and healthcare settings presents several challenges [3, 4]. Further challenges include the school nurses’ ability to clearly define their own professional roles within the school environment and clarify their own values and personal and professional philosophy [18].

Nursing theories inform knowledge development and promote theory-guided practice [19]. In the past, nursing science adapted theories from other disciplines, for example, education, psychology, sociology, and public health, and applied them to nursing [10]. Theories guide nurses in their understanding of the nursing roles and practices, helping them interpret their work comprehensively and insightfully, while also defining the scope of the nursing professions [2, 10]. Practice theories in nursing articulate the goals of nursing and outline the related care measures to achieve them [2]. Theories, models and frameworks organise existing knowledge and provide a more comprehensive picture of practices by integrating professional knowledge [1, 2, 10]. In addition, they support practice development and innovation, as well as decision-making [1, 2, 10]. Frameworks enable school nurses to focus on their missions and directions of their work, whereas predominantly task-oriented practice risks limiting their scope of practice [20]. Furthermore, models and frameworks provide a common understanding on what school nursing is, support school nurses’ role development and the communication of their work to internal and external partners [1, 5]. Theoretical foundations support practice implementation by explaining the underlying mechanisms of action and related successes or failures [21]. School nurses’ knowledge of theories and methods supports evidence-based and efficient health promotion [22].

As school nursing is a diverse field, a theoretical foundation provides a base for school nurses’ practice and an explanation of their professional context. To the best of our knowledge, there is no review of existing school nursing-specific models, frameworks and theories.

This scoping review aims to explore the conceptualisation and operationalisation of school nursing in theories, models and frameworks.

Our main question to be answered was: How is school nursing conceptualised and operationalised through theories, models and frameworks? To address this, we examined the key elements, objectives and tasks associated with school nursing, as well as the qualifications required for the role, the target populations. We also explored whether there were intended connections between school nursing and the health and education systems.

## Methods

The scoping review was conducted according to JBI Manual for evidence synthesis [23]. It is reported according to the Preferred Reporting Items for Systematic Reviews and Meta-Analyses extension for Scoping Reviews (PRISMA-ScR) Checklist [24] (Additional File 1). The prospectively developed protocol for the scoping review has not been published or pre-registered.

The following definitions served as the basis for this research. A theory is defined by Walker & Avant, 2014 as “an internally consistent group of relational statements that presents a systematic view about a phenomenon and that is useful for description, explanation, prediction, and prescription or control“ [2]. A model is defined by Bauer et al., 2015 as a “simplified depiction of a more complex world with relatively precise assumptions about cause and effect“ [25]. “Frameworks provide a broad set of constructs that organize concepts and data descriptively without specifying causal relationships” [25]. Concepts are described by Walker & Avant, 2014 as “basic building block of theories” [2], frameworks, and models [2, 10]. A conceptual framework, according to Huckabay, 1991, provides a systematic, unified way to guide nursing practice, education, and research [13, 26]. As the terms theory, (conceptual) model, and (conceptual) framework are often used interchangeably or imprecisely in nursing and/or implementation science [10, 25], we followed in most cases the terms used by the authors of included reports. We grouped models and frameworks in one category and reported them together. We used the term ‘theoretical foundations’ as a collective reference to theories, models, and frameworks in this scoping review.

### Eligibility criteria

We predefined eligibility criteria oriented on the Sample, Phenomenon of Interest, Design, Evaluation, Research type (SPIDER) terms [27]. We included theoretical foundations on school nursing in school setting, all school types and all countries. The phenomenon of interest were theories, models, frameworks and concepts describing school nursing. We included different sources of evidence including primary studies, textual papers, reviews as well as grey literature such as policies. There was no limitation applied to study designs, publication types, timeframe, country and language. We excluded individual interventions, campaign descriptions, legal laws unrelated to theoretical construct, reports on interprofessional collaboration, and publications on school health that did not describe school nursing. Articles referring to models or frameworks without detailed explanation were also excluded.

### Search and selection

We searched in the following databases: Medline/PsycInfo via OVID, CINAHL via EBSCOhost and ERIC via ProQuest from the earliest date until last search date, 08 March 2024. In addition, we screened the background and reference lists of the included reports to identify additional literature and supplement the search. We chose the search terms based on the inclusion criteria and a review of keywords in the previous literature. We used the Boolean operators “OR” and “AND” to build the search strings (Additional File 2), focused on title and abstract level.

### Selection of sources of evidence

**Fig. 1:**
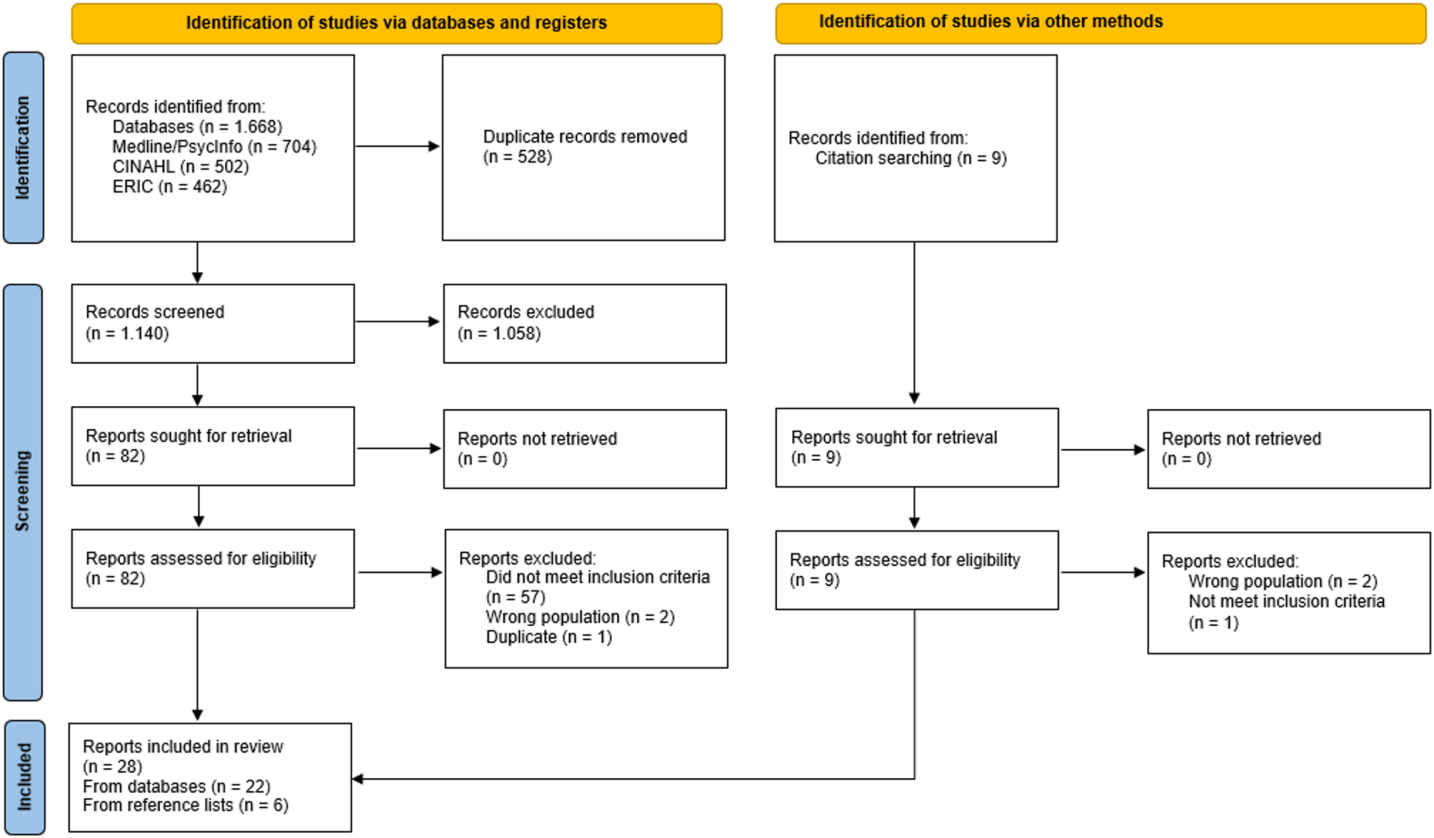
PRISMA flow chart retrieval and selection process of articles [31]

Two researchers (JK, AMP) screened the search results independently using Rayyan software [28]. First, we screened the titles and abstracts to include relevant records for full-text assessment. In a second step we retrieved the reports of selected records via the data bases, journals and online research, and read the included reports (Fig. 1). We resolved conflicts arising during the screening process based on consensus. No formal critical appraisal was applied.

### Data extraction and analysis

The data extraction sheet was developed based on the previously defined research questions (JK), discussed within the research team, and pretested with two reports by JK and AMP. Extracted data comprised theoretical approach, author, (model) name, publication year, country, publication title, development, setting, school type, target group, aim, definition of school nursing, tasks, qualification, key elements and funding/employment, practice application (if reported). If a model or framework was reported in more than one publication, it was only counted once. Refer to Tables 1 and 2 and Additional File 3 for details. The tasks were categorised deductively on the basis of the “Types of school health service (SHS) activity” of the World Health Organization (WHO) guideline on school health services [3] which included: (1) health promotion; (2) health education; (3) screening (leading to care and/or referral and support, as appropriate); (4) preventive interventions (such as immunizations and mass drug administration); (5) clinical assessment (leading to care and/or referral and support, as appropriate); (6) health services management; and (7) support for other pillars of a health-promoting school (HPS). Additional tasks identified in the included literature were inductively clustered. Information about the practical implementation of the theoretical constructs was extracted as secondary outcome, where available; however, the search strategy was not specifically tailored to identify this information.

Data extraction was conducted independently by two researchers (JK, AMP). Conflicts were resolved by discussion between two reviewers until a consensus was reached. Data were organised using Microsoft Excel Spreadsheet. We used elements of theory and concept analysis according to Avant & Walker [2] and content analysis according to Mayring [29] for data analysis to answer the following sub questions: (1) What key elements, tasks and goals are associated with school nurses/nursing? (2) Who is the target group? (3) What qualification is associated with the respective role? and (4) what connections are intended between school nursing and the health and education systems? The data extracted from the included papers were narratively summarised, common model components identified and frequencies of tasks counted to identify differences and similarities. The results were organised into descriptive tables. In the second step, content-based types were developed to synthesise and structure the identified models and frameworks [30]. Data synthesis was conducted by one researcher (JK) and reviewed through collaborative discussion within the research team to ensure consistency and rigor.

## Results

The database search yielded in 1.668 records, of which 528 duplicates were manually removed. After screening the remaining records, 82 reports were assessed for eligibility, resulting in the inclusion of 22 reports. Additional reference list and background screening resulted in nine reports, of which we included six. Reports excluded in the category “did not meet inclusion criteria” contain: already included models/frameworks/theories, interprofessional collaboration, single interventions, or school health without descriptions on school nursing. The total number of included reports is therefore 28, the PRISMA flow chart [31] is presented in Fig. 1.

Two of the included reports used both terms, concept and model [7], and were categorised to the model/framework section. Some of the 28 reports dealt with the same model or framework, so that a total of eleven different models, seven different frameworks and two theories were identified. The included reports were conducted in Canada (*n* = 2), Colombia (*n* = 1), Republic of Korea (*n* = 1), Sweden (*n* = 1), United Kingdom (*n* = 5), United States of America (*n* = 17) and one in the United Kingdom & the United States of America.

An overview of the theories, models and frameworks and related key elements/contents is provided in Additional file 3. Table 1 provides information on author, country, year, development, practice application, setting and school type, school nursing aim, definition and target groups. Table 2 includes information on school nurses’ qualification, tasks and collaboration.

### Theories

Two theories with concrete references to school nursing were identified, a holistic theory of Garmy et al., 2021 [18] and a situation-specific theory of Broussard, 2007 [16]. Both theories include person-centred elements. Garmy et al. [18] focusses on the integration of values in school nursing practice, while Broussard [16] focusses on the empowerment in school nursing practice. The school nurses play a key role in health promotion and disease prevention, involving complex interactions with students and the (school) environment [18]. To face these challenges and needs, the school nurses’ role under the given working environment entail clarifying the values and philosophy, Garmy et al. adopted “Barbara Dossey’s holistic nursing theory on school nursing” [18]. Holistic nursing is ”*founded on the values of integrality and awareness of whole-people and whole-system interconnectedness“* [18]. Nurses have professional and social competences, and all five components of Barbara Dossey’s holistic nursing theory are connected to person-centred school nursing [18]. These five components include healing, the metaparadigm of nursing theory, patterns of knowing, the four quadrants (internal & external individual factors, internal & external collective factors) and a conclusion of all components [18].

School nurses need a sense of empowerment to foster their own professional development and achieve their goals, which enables them to provide the level of care expected by the school community and other professionals [16]. Therefore, the situation-specific theory, developed by Broussard, 2007, “Making A Difference: The School Nurse’s Role in the Health of Children in the School Setting” [16] focusses on the empowerment process in the practice of school nurses. It consists of four theoretical constructs, which influence the empowerment in school nurses practice in different ways: (a) enlisting support, (b) getting through the day, (c) maintaining control over practice, and (d) adjusting to challenges [16].

### Model and framework - types

The identified models and frameworks have a varying focus within the overarching theme “school nursing” and were clustered in “Role and practice models/frameworks”, “Organisation and delivery models/frameworks” and “Qualification models/frameworks”, see Table 1.

#### Role and practice models/frameworks

Practice frameworks aid school nurses “in explaining and accomplishing their role” [32]. Role and practice models and frameworks provide key answers to the questions of what school nursing is, the definitions of school nursing, school nurses’ roles with the focus areas and goals of their work and the related tasks. This category includes eleven models and frameworks [1, 7, 13, 33–47], see Table 1.

#### Organisation and delivery models/frameworks

This category is oriented towards health service delivery model, including its components and interacting elements [48]. It includes models and conditions answering how school nursing is organised or delivered, access, funding, employment/government functions as well as, regulations and laws. Five models and frameworks were assigned to this category [5, 14, 49–52], see Table 1.

#### Qualification models/frameworks

This category subsumed models and frameworks with information on school nurse’s qualification and associated skills and competencies required for their tasks. One model and one framework were clustered to this category [15, 53], see Table 1.

**Table 1:** Theories, models and frameworks on school nursing-summary

First Author Year Country	Name	Development*	Practice application**	Setting/ School Type	Objectives	Definition school nursing	Target group
Theory							
Garmy, P. 2021 [18] Sweden	School Nursing Framed by the Holistic Nursing Theory	Theoretical	-	School/ no special type reported	By working on the basis of Barbara Dossey’s holistic nursing theory, the school nurse is given tools to deal with phenomena such as people’s experiences, feelings, and needs.	The school nurse promotes health and prevents disease.	Students
Broussard, L. 2007 [16] United States of America	Making a Difference: The Role of the School Nurse in the Health of Children in Schools	Theoretical, empirical	-	School/ no special type reported	Empowerment process in the practice of school nurses	-	Students, families, teachers
Role- and practice models/frameworks							
Lewallen, T. 2015; Galemore, C. 2016; Driscoll, L. 2021 [33–35] United States of America	Whole School, Whole Child, Whole Community	Empirical	Yes	School/ no special type reported	Systematic, integrated, and collaborative approach to health and learning, supported by school environment	-	Students/ child
Joint Consortium for School Health 2008, 2016; Buduhan V.H. 2021 [13, 36] Canada	Comprehensive School Health Framework	Theoretical	Yes	School/ Public Health / no special type reported	Common goal of supporting the educational and health outcomes of children by building healthy school communities	Schools are one practice setting of public health nursing in Canada, nurse leaders need to demonstrate how a public health nurse can fit into the existing national framework Comprehensive school health	Students
Ferro, M.C. 2020 [37] Colombia	Model of professional practice of school nursing for Colombia education and health: a care bond	Empirical	Practice validation	School/ no special type reported	Provide quality care to children, generating a caring bond, which unites education and health, propitiate the inclusion of children or adolescents, facilitating all to develop their school activities	School nursing requires generating a caring bond, which unites education and health. For this, both the school environment and the kind treatment by the nurse are fundamental to establish a relationship of trust that favours caring for the members of the education community.	Students and their school community
NHS Wales 2017 [47] United Kingdom, Wales	A Framework for a School Nursing Service for Wales’	-	Yes	School/ Public Health / Primary & secondary schools	School nurses have a designated role as a key contributor to the early years’ development of a child which targets the health and emotional needs of school aged children and young people.	School nurses as Specialist Community Public Health Nurses (School Nursing) graduates are qualified to provide holistic, individualised community and population level public health services.	Students (families)
Maughan, E. 2015; 2016; National Association of School Nurses 2018, 2020 [1, 38–41] United States of America	Framework for 21st Century School Nursing Practice™	Empirical	Yes	School/ no special type reported	Keeping students healthy, safe, and ready to learn	School nursing practice occurs along several continuums: individualized care to population health, generalist to specialist practice, and health promotion to disease prevention. Although school nursing remains rooted in its founding activities of acute care, health education, and communicable disease, school nursing has also changed over the years in order to meet the evolving health needs of students.	Student (family, community)
Brooks, F. 2007 [7] United Kingdom; (United States of America)	The school nurse as navigator of the school health journey	Theoretical	-	School/ Public Health/ no special type reported	School nursing may offer a vehicle for the delivery of an effective public health strategy for children and adolescents	The school nurse is the only professional concerned with children’s well-being that traverses all the environments of the child, that is, the home, the school and the wider community as well as connecting with the multi-sectoral nature of the service provision for young people.	Children and adolescents
Hilsinger, G. 2006 [42] United States of America	Broad Scope of School Nursing Practice	-	Yes	School/ Public Health/ no special type reported	Universal and targeted activities, directed to all students to create a supportive learning environment for every student and concentrated in the few students with identified problems	School nurses make to improve student health and well-being, which leads to educational success.	Students
Wicklander, M.K. 2005 [43] United Kingdom	National Healthy School Standard framework (NHSS)	-	Yes	School/ Public Health/ all types of schools	Aims of the NHSS are to reduce health inequalities, to promote social inclusion, and to raise educational standards. Analyse the school nursing role within the NHSS with an aim of increasing awareness of the framework as an effective tool for strengthening the role of school nurses globally.	School nurses have the clinical expertise and knowledge to support school staff in the whole-school approach; assessing the health needs of the school population; link schools with primary care services.	Students (and staff)
Barnfather, J. S. 1991 [44] United States of America	Modeling and role-modeling - Restructuring the role of school nurse	Theoretical	Yes	School/ High schools	School nurses can systematically facilitate years of schooling in this population who are at risk for impaired growth, development, and health.	School nursing practice is based on theory-driven nursing process, modeling and role-modeling as theoretical framework.	Students
Rustia, J. 1982 [45] United States of America	Rustia School Health Promotion Model	-	Yes	School/ no special type reported	Goal: promoting optimal health. objectives: primary, secondary and tertiary prevention.	The model uses nurses as the health service providers responsible for implementing the program.	Students (family)
Wold, S.J. 1979 [46] United States of America	School Nursing - A Framework for Practice	Theoretical, experts	-	School /Public Health/ no special type reported	School nurse specifically focuses on promotion of “high level wellness”, maximizing the potential of which the individual is capable within the environment where he is functioning	School nursing is a part of community nursing and therefore has as its basis an understanding of ‘‘public health.”	Student (family and school community)
Organisation- and delivery models/frameworks							
Wheeler, B.A. 2017, 2019 [49, 50] United Kingdom	Berkshire school nursing team’s innovative service model	-	Yes	School/ Public Health/ all types of schools	Raising visibility of school nurse service thus improving direct access for our school-aged children, young people and families to access early help from a school nurse they knew and trusted.	School nurse understanding and requirement based on “healthy child programme” Department of Health,	Students
Becker S.I. 2017 [51] United States of America	Several school health delivery models	Empirical	Yes	School/Public Health/ no special type reported	School nurses deliver and improve access to care to children, across the nation, on an almost daily basis; school nurses are central to the delivery of public health agenda	School nursing understanding as universal service -focus on the student (and community for assessments)	Student (family)
DH CNO Professional Leadership Team 2012 [5] United Kingdom	A service model for school nursing	-	Yes	School/ Public Health / no special type reported	Goal: reach and support the health and wellbeing of school-aged children	School nursing contribution to improving and protecting health.	School aged children and young people
Sheetz, A.H. 2003 [14] United States of America	Enhanced School Health Service (ESHS) Model	-	Yes	School/ Public Health/ no special type reported	Goal of the ESHS-program: is to provide all school-age children, in schools throughout the Commonwealth of Massachusetts, access to a school health service program that is: Community based. Integrated within and supportive of the educational system. Managed by a qualified nursing leader	School nursing has its roots in public health nursing and remains a public health specialty. […] From these early beginnings in infection control, school nursing has developed into a comprehensive specialty with an array of prevention and treatment responsibilities.	Students (families, community based)
Wisconsin State Legislative Council 1994 [52] United States of America	School Health Service Delivery in Wisconsin	-	Yes	School / Public Health/ no special type reported	Guarantee that a basic educational opportunity is available to all pupils regardless of the local fiscal capacity of the district in which they reside.	-	Students and staff;
Qualification models/frameworks							
Shin, E.M. 2020 [53] Republic of Korea	School nurse competency framework for continuing education	Empirical	-	School Nurse Education/ all types of schools	The framework provides a basis for developing both a continuing education curriculum for the professional development of school nurses and standards of school nursing practice. School nurses play an important role in the school setting and are responsible for the health of students with diverse healthcare needs	School nurses’ six core competencies include the ability to (1) provide patient-centered care through the integration of knowledge and skills; (2) communicate and collaborate with students, teaching staff, and community resources; (3) think critically for evidence-based practice; (4) implement school health services and programs; (5) integrate legal and ethical nursing practice; and (6) conduct health education.” school nurse competencies are the knowledge, skills, and attitudes required of school nurses to provide safe school healthcare.	Of the school nurse: students; of the education framework: school nurses
Keller T. 2004 [15] United States of America	Differentiated Practice Model for School Nursing	-	-	School (& Public Health)/ no special type reported	This proposed model is based on differentiation by education as this appears to be the best method of matching nursing preparation to an expanded professional role; effectively using available school nurse resources by spreading role responsibilities across three levels.	Autonomous nursing specialty, the role of the school nurse has expanded from strictly public health function	Student, family, school/ wider community, district/region; state offices ^y)^

* If reported, development was categorized in theoretical and empirical

** not primary endpoint, extracted if reported (- = unclear/not reported)

y) dependent on their qualification-level, school nurses have different tasks and target groups

ESHS = Enhanced School Health Service; NHSS = National Healthy School Standard framework

### Definitions of school nursing, school nurses’ role and related objectives and goals

Not all of the included publications reported a definition of school nursing, while some described the roles, aims and goals, with different levels of details (Table 1). A school nurse was defined as an autonomous professional responsible for the health of students at schools, and providing safe healthcare in school, and meeting the evolving health needs of students [1, 5, 14, 15, 18, 45, 49, 53]. This goes beyond traditional public health roles of school nurses. Other authors focused more on the combination of health and education, as a universal or comprehensive service for the school community [14, 37, 42, 51]. School nurses were seen as experts in providing the public health services, on a holistic, individualised level for different communities and populations [13, 15, 36, 46, 47]. Furthermore, they support school staff in the whole-school approach, with assessing the health needs of the school population and linking schools with primary care services [43]. The following definition, given by Brooks et al., 2007, goes beyond the previous definitions by connecting all student environments, *“the school nurse is the only professional concerned with children’s well-being that traverses all the environments of the child*,* that is*,* the home*,* the school and the wider community as well as connecting with the multi-sectoral nature of the service provision for young people”* [7]. Barnfather, 1991 described that school nursing practice was based on the theory-driven nursing process, framed by modeling and role-modeling [44].

The objectives of the theories, models and frameworks and related target group differed and were therefore clustered in “objectives related to students” and “other objectives”.

#### Objectives related to students

Most of the aims were **education support** (by addressing health needs) [1, 33, 35–37, 42, 52] as well as **improving or supporting the health of students** [5, 46, 47, 52, 53]. Further objectives were **delivering quality (health) care** [37, 49], **improving access to health care** [14] or **both combined** (daily provided) [1, 51]. Further reported objectives include **providing/delivering public health agenda** [7, 51] and **health promotion** (preventive health care) [5, 45]. Each one aimed (also) on **school attendance** [44] and **inclusion of children** [37]. Other strategic aims were to **reduce health inequalities**, to promote **social inclusion**, and to **raise educational standards** with the key goal to have access to a school nurse for every school in the United Kingdom (by March 2006) [43]. Universal and targeted activities aimed at all students regardless of whether they have special health needs or not, to create a supportive learning environment [42].

#### Other objectives

Garmy et al.’s theory aims at empowering the school nurses to effectively deal with different phenomena (people’s experiences, feelings, needs) [18]. The model of Keller et al., 2004 aims at an effective use of available school nurse resources by distributing roles and responsibilities according to the three qualification levels for school nurses (Bachelor’s degree, Master’s degree and PhD) [15]. The framework of Wicklander, 2005 aims to strengthen the role of school nurses globally [43]. Shin & Roh’s framework (2020) aims with focuses on continuing education curriculum aimed at the professional development of school nurses and standards of school nursing practice [53].

### Setting and school type

Settings that have been defined as the school nurse’s field of action are the school exclusively [16, 18, 33, 37, 44, 45] or school and public health service (in the community) [1, 5, 7, 14, 15, 36, 42, 43, 46, 47, 49, 51, 52]. One framework does not focus on the working field but rather on the educational setting for school nurses [53]. Most theoretical foundations do not refer to a special school type [1, 5, 7, 14–16, 18, 33, 36, 37, 42, 45, 46, 51, 52], or include all school types [43, 49, 53]. Only two specifically focussed on high schools [44] or primary schools and secondary schools respectively [47].

### Target group

The primary target group of school nurses in all theoretical foundations is students (children/adolescents). To address students’ needs, students must be viewed in the context of their environment, as it shaped and influenced them. Related to this understanding, the (school) community and students’ families are seen as additional target groups [51]. Some school nurses extend services beyond the school and targeting the broader communities [14, 15, 51]. The extent or dimension of care for the school community, other communities and families are not described in details. School nurses themselves are the target group of the education framework [53]. The target groups of school nurses also vary to some extent based on their qualification: the Generalist Bachelor of Science in Nursing (BSN) focusses on students, families and the school communities; the Master’s-prepared school nurse (MSN) serves the wider community, district or region; and the school nurse analyst (PhD) is responsible for school health services located at state offices [15].

### Tasks

Table 2 gives an overview of the different tasks. Tasks that did not fit into the predefined categories of the WHO guideline on school health services [3], were summarised in an additional category “others”.

(1) **health promotion** is described most often, by 18 different reports included in the review [1, 5, 7, 14, 18, 33, 36, 37, 42–47, 49,51–53]; (2) **health education** is reported by 17 reports [1, 5, 7, 14, 15, 18, 33, 36, 37, 43, 45–47, 49, 51–53]; (3) **screening** (leading to care and/or referral and support, as appropriate) is reported by 11 reports [1, 5, 14, 15, 36, 42, 45–47, 51, 52]; (4) **preventive interventions** (including immunisations, mass drug administration) are reported by 13 reports [1, 5, 7, 14, 18, 36, 37, 42, 45–47, 51, 52]; (5) **clinical assessment** (leading to care and/or referral and support, as appropriate) is reported by 15 [1, 5, 7, 14, 15, 33, 37, 42, 45–47, 49, 51–53]; (6) **health services management** is reported by eight [14, 33, 36, 37, 42, 43, 49, 53]; (7) **support for other pillars of a HPS** is reported by seven [14, 36, 37, 43, 45, 49, 53]. (8) **others**, added from the literature, **leadership** (including programme management, decision making) was reported by eight [1, 7, 14, 15, 45, 47, 49, 52]; five reported service/care coordination (guidance service, transition to adult services) [5, 7, 15, 36, 49]; each three of the included reported evaluation [15, 45, 52], education for school or health personnel [13, 15, 43] and health information offers/access (for families) [7, 43, 47]. Each two of the included reports highlighted counselling/supervision [15, 33], community work [46, 51], supportive school environment [37, 42] and self-care/self-assessment [7, 43], and one reported tasks to support school attendance [44]. Keller & Ryberg [15] differentiate the tasks of school nurses according to their qualification level (BSN, MSN and PhD), see Table 2.

**Table 2:** Theories, models and frameworks - school nurses‘ qualification and tasks

Autor	Qualification	1. Health promotion	2. Health education	3. Screening	4. Preventive interventions	5. Clinical assessment	6. Health services management	7. Support for other pillars of a HPS	8. Other	Cooperation/Collaboration
**Theory**										
Garmy [18]	-	X	X	?	X	?	?	?	-	-
Broussard [16]	School nurses were not required to hold a bachelor’s degree, but it is recommended	?	?	?	?	?	?	?	-	-
**Role- and practice models/frameworks**										
Lewallen; Galemore; Driscoll [33–35]	No information	X	X	?	?	X	X	?	Counselling	Collaboration needed among school, health, and community sectors; school social worker, school psychologists
Joint Consortium for School Health 2008, 2016; Buduhan V.H. 2021 [13, 36]	Public health nurse has a bachelor’s degree in nursing and is a member of a professional regulatory body for registered nurses ^d)^	X	X	X	X	-	X	X	Guidance service; ^d)^ health education support for teachers	
Ferro [37]	-	X	X	?	X	X	X	X	School environment	Cooperation with other colleagues
NHS Wales [47]	Registered school nurse, Specialist community public health nurse	X	X	X	X	X	?	?	Leadership for the school nursing team; parents evenings offer information and public health messages	Public health service, school nursing team; community dental services;
Maughan 2015; 2016; National Association of School Nurses 2018, 2020 [1, 38–41]	-	X	X	X	X	X	?	?	Leadership	-
Brooks [7]	Nurse, no further information	X	X	?	X	X	?	?	Coordination of care; Information; Decision making; Self-care; leadership	-
Hilsinger [42]	-	X	?	X	X	X	X	?	Supportive learning environment	-
Wicklander [43]	-	X	X	?	?	?	X	X	Education support, access to health information, school nursing self-assessment	Network and local cooperation
Barnfather [44]	-	X	?	?	?	?	?	?	Tasks to support school attendance	-
Rustia [45]	Nurse, no special information	X	X	X	X	X	?	X	Evaluation, leadership	School health team; teachers, supportive personnel, community;
Wold [46]	Nurse	X	X	X	X	X	?	?	Home visits	Health team coordinator
**Organisation- and delivery models/frameworks**										
Wheeler [49, 50]	Specialist community public health nurse no further information;	X	X	?	?	X	X	X	Leadership, transition to adult services	School health team
Becker [51]	Registered nurse (RN) most master qualified: focus on tasks only the RN can do. Sometimes together with a licensed practical nurse, Unlicensed assistive personnel (UAP).	X	X	X	X	X	?	?	Community work to identify families’ needs	Community navigators; depending on the funding/employer: local hospitals, public health departments, and government agencies;
DH CNO Professional Leadership Team [5]	Qualified nurses or midwives with specialist graduate level education in community health	X	X	X	X	X	?	?	Service coordination	Interprofessional cooperation
Sheetz [14]	BSN- or MSN and community health, school health, or paediatric experience ^c)^	X	X	X	X	X	X	X	Leadership	Community; coordinate a wide spectrum of school and community resources and services;
Wisconsin State Legislative Council [52]	Registered nurse, licensed, certified by the DPI	X	X	X	X	X	?	?	Evaluation, program and data management	Professionals and community;
**Qualification models/frameworks**										
Shin [53]	Job training as “a teacher” and continuing education as “a registered nurse”	X	X	?	?	X	X	X	Tasks derived from the competencies	Collaborate with teachers, medical advisors, and stakeholders*
Keller [15]	1st Generalist (BSN); 2nd Master’s Prepared School Nurse ^a);^ 3rd School Nurse Analyst (PhD) ^b)^	?	X, a, b	X, a, b	?, a	X, a, b	?, a, b	?, a, b	Education and supervision for assistive personnel; ^a)^ leadership, coordination and evaluation of (school) health services, consultation for BSN/ Nurses; ^b)^ developing and evaluating	School nurses’ collaboration between all educational levels; different communication partner; dependent on education level; ^a)^ local stakeholders*, school nurses;

X = yes;? = unclear/not described; - = no

^a)^ more district level, coordination of the different tasks, (health) education for health services personal and district/region, direct care in complex cases

^b)^ policy and state level, developing and evaluation of the programmes

^c)^ Bachelor’s or Master’s degree in nursing & community health, school health, or paediatric experience to manage school health program; BSN- or MSN-prepared professional nurses to care for students and families, no further differentiation in tasks between education level reported

^d)^ Described by Buduhan et al. 2021 [13]

* Following the authors Akl, E. et al. 2024 [60], we use the term interest-holder instead of stakeholder. In the tables, the original terms from the respective publication are used

BSN = Bachelor of Science in Nursing; DPI = Department of Public Instruction; HPS = Health-promoting school; MSN = Master of Science in Nursing; NHS = National health service; PhD = Doctor of Philosophy; RN = Registered nurse; UAP = Unlicensed assistive personnel

### Cooperation and collaboration

School nurses collaborate and cooperate with different persons inside and outside the school. Within the school, these include teachers, supportive personnel or school health professionals (e.g. school psychologists, social worker) [14, 33, 37, 45, 49, 52, 53]. Outside the school, they collaborate with local interest-holders [15, 43, 53], the community [14, 33, 43, 45, 52], public health services and medical advisors [47, 53] and different funders/employers (e.g. local hospitals, public health departments, health team coordinator) [46, 51]. In addition, intraprofessional collaboration between school nurses was reported [5, 15, 47].

### School nurses’ qualification

Overall, a Bachelor’s degree in nursing/registered nurse is recommended as the minimum qualification for employment as school nurse [15, 16]. The reasons given are the minimum competences required for their tasks, but also a lack of adequately qualified staff with a Master’s degree [15]. Further mentioned qualifications are a Bachelor’s degree with additional training in community or school or public health nursing [5, 13, 14, 47, 49, 52] or a Master’s degree in one of these fields [14, 15, 51]. One of the included reports requires a qualification as nurse or midwife [5] and one a double qualification as registered nurse and teacher [53]. Other sources included in the review, particularly older literature, reported a (registered) nurse qualification without further information [7, 45, 46].

### Funding and employment

Employment and funding differ, in alignment with the respective health and/or education system of the country. The funding for school nursing is mainly sourced from the health or education sector [52], or from a combination and collaboration between both [43, 51]. The health sector in this context includes the health department, public health or local hospitals/health care systems [7, 51]. As a special form of funding from the health sector, the use of the tobacco tax was described as another source for school nursing [14]. Further funding sources are government (agencies), private insurances, foundation grants [13, 36, 51], private schools [37], or a mix of resources (e.g. between provinces) [13, 49, 50]. Related employers in the education sector are school districts [15, 52], and in the health sector, the local health departments and local National Health Service (NHS) [15, 47, 52]. Additionally, Wheeler, 2017 & 2019 reported different employers without further explanation [49, 50].

### Practice application

Out of 13 models and frameworks practice application, or pilot testing with subsequent continuation were reported [5, 14, 34, 36, 40, 42–45, 47, 49, 51, 52], one reported validation of practice application [37].

### Phenomena of trust

Several theoretical foundations included the phenomena of trust. The school nurse is seen as a trusted person for students and their community, and colleagues [16, 46, 50] or as a person who has to establish/build a relationship of trust [37, 44]. In the context of delegation, school nurses need to trust those to whom they delegate their tasks [16].

## Discussion

Different theories, models and frameworks for school nursing were identified. Most of them were published from 2012 onwards, with the earliest dating back to 1979 by Wold and Dagg [46]. As is common in the field, existing theoretical foundations from various disciplines or nursing contexts [10] were adapted to the school nursing context, e.g. “Holistic nursing theory on school nursing” by Garmy et al. [18] and “Modeling and role-modeling Restructuring the role of school nurse” by Barnfather [44]. The interchangeable use of the terms model and frameworks has also been demonstrated in the included reports. However, an example for applying the differentiated terminology is the “Whole School, Whole Community, Whole Child” model [33], conceptually specified by the “Framework for 21st Century School Nursing Practice” [1], with different interacting concepts.

The understanding of school nursing varies across theoretical foundations. Some reports offer only aims and objectives rather than an explicit definition. Therefore, the aims and definition of school nursing together create a more complete picture. The scope of school nursing has expanded over the years from traditional physical health care activities with screening and first aid to comprehensive health care interventions, addressing the unmet health care needs of students [45]. Even though the definitions of school nursing vary, a common understanding and goal setting of all interest-holders/parties involved in health and education in the school setting is important to reach the desired aims. According to the aims, the role of school nurses, their target group, necessary offers, tasks, collaboration, qualification and subsequent financing and structural integration needs to be clearly defined. The school environment is seen as a supporting element in a collaborative approach to health and learning [33, 35]. School nurses primarily target students, but also support school staff, families, and to some extent community outpatient services. Therefore, they cooperate with different parties and build a supporting network [33]. School nurses collaborate with other professionals and are integrated into the school team, yet they mostly remain the only onsite representatives of their profession. Their work integrates elements from various fields, and its distribution across health and education sectors might be a source of tension or conflict [3]. School nurses face expectations regarding their (leadership) role within and outside the school, requiring orientation to foster a shared (self-)understanding [4, 16]. Therefore, intraprofessional collaboration, cooperation and supervision could support practice by fostering role development, particularly for novices in the field. This support contributes to addressing challenges related to their independent practice settings and roles, such as feelings of isolation and uncertainty [17]. However, this support was only described by three of the included reports [5, 15, 47].

In addition to the school health tasks described in the WHO guideline on school health services [3], leadership, evaluation and coordination were identified as key elements of school nursing, provided by school nurses. This demonstrates a complexity that goes beyond taking on single tasks and measures, rather an emphasis on individual prioritisation and associated autonomy. However, it remains unclear at this point to what extent school nurses are guided by others. Nonetheless, the frameworks guiding school nurses need to be broad enough to allow flexibility, individual prioritisation and goal setting, depending on the target group, school needs and the school nurse’s strengths.

Overall, school nurses require appropriate skills to provide target group specific health education offers, or depending on the complexity of situations and care offered, advanced nursing practice skills. Leadership requires necessary management competencies, as it includes developing visions and creating strategies, leading people, inspiring and motivating a team, and making decisions, to achieve common goals and driving innovation. Furthermore, school nurses hold a key position in developing and coordinating services and care; which includes organising, facilitating communication among different parties and overseeing the quality of services provided. In order to cope with the previously described diverse challenges and to navigate the continuously evolving field of practice - while also considering their own professional development - school nurses require competencies in self-care. These competencies were added in the updated School Nursing Practice Framework™ in 2024 [32]. In summary, the required competencies for school nursing go beyond a basic nursing qualification. The minimum qualification at Bachelor’s level is not only justified by the required competencies to fulfil the defined tasks and to achieve the goals, but also to gain acceptance and respect from other professions such as teachers [16]. A qualification on Master’s level, continuing education and evidence-based practice are described as necessary in some of the included theoretical foundations [14, 15, 51, 53]. The results give an overview of existing employment and funding resources, which are dependent on and in alignment with the respective health and/or education systems of the country.

In professional practice, theoretical foundations are useful for developing key questions, guiding observations and data collection, interpreting data and making decisions to address students’ needs [10, 32, 50]. Nevertheless, the motivation to develop models differs and ranges from role descriptions, addressing system-related challenges [49], to limited personnel resources [15]. A clear (service) model linked to good health outcomes is needed to inform future commissioning [5]. For example the “Framework for 21st Century School Nursing Practice” [1] was applied for school nurse job descriptions for “urban school districts in the United States” [54]. This framework also guided the development of an evaluation process which facilitates the growth of a school nurse and provides a structure for meaningful evaluation to better understand school nursing practice [55]. Societal transformation processes are also reflected in school nursing. Practice frameworks need to be updated continuously, as new evidence exists and standards of care change [32]. Therefore, the “Framework for 21st Century School Nursing Practice” [1] was updated in May 2024 to the “School Nursing Practice Framework™” [32, 56].

Barriers and facilitators of the implementation of school nursing were described by Wicklander, 2005 [43]. Barriers include the required time for the government’s immunisation programmes, a limited understanding of the school nurses’ role in bridging the health and education agendas, with limited recognition of school nurses’ role in supporting educational attainment, a lack of investment in professional training and development, and varied skills, capacity, and workloads of school nursing services across the country. Facilitators include positive relationships between school nursing services and others, accessible and adequate funding to local health and education partnerships, support and mentorship for school nurses; supportive leadership, available resources, and access to continuing education courses [43].

In conclusion, there is no universal model or framework of school nursing, each has according to its intention benefits and complements the others, illuminating specific aspects. We categorised the various models and frameworks based on their primary objectives and areas of focus into different types. However, the categorised models and frameworks should not be seen as entirely distinct, as they contain overlapping elements. For instance, the qualification models and frameworks also include information on the school nurses’ tasks and related required competencies. Moreover, the different model/framework types show the complexity of school nursing. The information and contextual factors included in the different types might support the practice, implementation and evaluation [57, 58]. The models and frameworks could provide a basis for necessary qualification, qualification related tasks, task-related funding resources, employment, goal setting and evaluation. The differences in the models and frameworks show that the tasks, scope and collaboration of school nursing practice also depend on the overall approach of the health care and education system with related aims, funding and resources. In summary, the different models and frameworks together could serve as a solid basis and inform the implementation of school nursing in alignment with the respective health care system and structures, as a complex intervention [57–59].

Both identified theories on school nursing [16, 18] are guided by a holistic and person-centred perspective and aim to support school nurses through value clarification, professional orientation and empowerment, regardless of the school nursing practice approach.

### Strengths and limitations

A wide variety of models, frameworks and theories for school nursing have been described and important contextual factors regarding the implementation of school nursing were summarised. We did not limit the analysis to countries and analysed the included reports systematically; however, most were developed in the United Kingdom and the United States of America. We did not limit the publication date. Our motivation to include reports over the whole time period was to gather information on how long models have been available. In addition, older theoretical foundations may also offer hints for current school nursing practice or its development. The level of detail between the included reports differs, as the theories and models have different levels and purposes resulting in no in-depth description of individual tasks. However, more structured details would probably lead to an even more concrete overall view. Although the search strategy was developed in consultation with experienced researchers, the absence of a professional information specialist may have reduced the sensitivity of the search. Additional (grey) literature included was searched via reference screening, beyond this no further efforts have been made. The data analysis was conducted by only one researcher. However, the results were discussed with a supervising researcher.

Further research on the practice implementation and evaluation of the models and frameworks could provide additional information, especially on barriers and facilitators for practice implementation, and is therefore needed.

## Conclusions

This scoping review provides an overview on existing models and frameworks of school nursing including hints on diverse and useful approaches. Next to health related tasks described in the WHO guideline of school health services [3], school nurses assume an autonomous key role including leadership tasks. These complex role profiles require appropriate competencies that go beyond the Bachelor’s degree level. School nursing is a continuing developing field. This is demonstrated by the included models and frameworks, which have been continuously developed over the entire period. The provided database on existing theoretical foundations for school nursing could inform practice implementation and development. Additionally, the theoretical foundations and results could guide further research by reflecting and evaluating existing practice in a systematic way. Theoretical foundations aim to support school nurses in the development of their roles by providing a base for their activities and a structure connected to the targeted aims. This is linked to the opportunity of evaluating the achievability of the goals under the given circumstances of the health care and education system and related funding resources. For countries without widespread implementation of school nursing, the identified models, theories and frameworks could serve as a basis and might support further implementation steps.

## Supplementary Information

Below is the link to the electronic supplementary material.

Supplementary Material 1

Supplementary Material 2

Supplementary Material 3

## Data Availability

The datasets analysed during the scoping review study (i.e., the raw coded data extracted from identified papers) are available from the corresponding author on request.

## Abbreviations

BSN: Bachelor of Science in Nursing
DPI: Department of Public Instruction
HPS: Health-promoting school
MSN: Master of Science in Nursing
NASN: National Association of School Nurses
NHS: National Health Service
PRISMA-ScR: Preferred Reporting Items for Systematic Reviews and Meta-Analyses extension for Scoping Reviews
PhD: Doctor of Philosophy
PHN: Public health nurse
RN: Registered nurse
SCPHN: Specialist community public health nurse
SPIDER: Sample, Phenomenon of Interest, Design, Evaluation, Research type
UAP: Unlicensed assistive personnel
UK: United Kingdom
USA: United States of America
