# Supplementary material for: Theories, models and frameworks of school nursing - a scoping review
Source: BMC Nurs. 2025 Sep 10;24:1164. doi: 10.1186/s12912-025-03730-5 (PMC12424212; doi:10.1186/s12912-025-03730-5)
Supplement: Supplementary file 2 — Supplementary Material 2 [file 12912_2025_3730_MOESM2_ESM.pdf]

Additional file 2 - Searchstrings

Medline & PsycInfo via Ovid

(((„SCHOOL NURS*“ or „SCHOOL-NURS*“) adj10 MODEL*).tw.) or (((„SCHOOL NURS*“ or „SCHOOL-NURS*“) adj10 DESIGN*).tw.) or (((„SCHOOL NURS*“ or „SCHOOL-NURS*“) adj10 APPROACH).tw.) or (((„SCHOOL NURS*“ or „SCHOOL-NURS*“) adj10 CONCEPT*).tw.) or (((„SCHOOL NURS*“ or „SCHOOL-NURS*“) adj10 FRAMEWORK*).tw.) or (((„SCHOOL NURS*“ or „SCHOOL-NURS*“) adj10 THEOR*).tw.) or (((„SCHOOL NURS*“ or „SCHOOL-NURS*“) adj10 SCHEME*).tw.) or (((„SCHOOL NURS*“ or „SCHOOL-NURS*“) adj10 HYPOTHESIS).tw.) or (((„SCHOOL NURS*“ or „SCHOOL-NURS*“) adj10 „THINKING MODEL*).tw.) or ((„SCHOOL NURSES“ adj10 (METHODOLOGY or THEORIES or MODELS)).sh.

ERIC via ProQuest

(abstract((„SCHOOL NURS*“ OR *SCHOOL-NURS*“) AND abstract((MODEL* OR DESIGN* OR APPROACH OR CONCEPT* OR FRAMEWORK* OR THEOR* OR SCHEME* OR HYPOTHESIS)))

OR (title((„SCHOOL NURS*“ OR *SCHOOL-NURS*“) AND title((MODEL* OR DESIGN* OR APPROACH OR CONCEPT* OR FRAMEWORK* OR THEOR* OR SCHEME* OR HYPOTHESIS))) OR (mainsubject((„School Nurses“)) AND mainsubject((„Models“ OR „Theory Practice Relationship“ OR „Generalizability Theory“)))

Cinahl via Ebsco

((TI „SCHOOL NURS*“ OR AB „SCHOOL NURS*“) N10 (TI MODEL* OR AB MODEL*)) OR ((TI „SCHOOL NURS*“ OR AB „SCHOOL NURS*“) N10 (TI DESIGN* OR AB DESIGN*)) OR ((TI „SCHOOL NURS*“ OR AB „SCHOOL NURS*“) N10 (TI APPROACH OR AB APPROACH)) OR ((TI „SCHOOL NURS*“ OR AB „SCHOOL NURS*“) N10 (TI CONCEPT* OR AB CONCEPT*)) OR ((TI „SCHOOL NURS*“ OR AB „SCHOOL NURS*“) N10 (TI FRAMEWORK* OR AB FRAMEWORK*)) OR ((TI „SCHOOL NURS*“ OR AB „SCHOOL NURS*“) N10 (TI THEOR* OR AB THEOR*)) OR ((TI „SCHOOL NURS*“ OR AB „SCHOOL NURS*“) N10 (TI SCHEME* OR AB SCHEME*)) OR ((MH „SCHOOL NURSES“ OR MH „SCHOOLS, NURSING“ OR MH „SCHOOL NURSING“) N10 (MH THEORY OR MH „MODELS, THEORETICAL“ OR MH „NURSING THEORY“ OR MH „CONCEPTUAL FRAMEWORK“))
