# Supplementary material for: Theories, models and frameworks of school nursing - a scoping review
Source: BMC Nurs. 2025 Sep 10;24:1164. doi: 10.1186/s12912-025-03730-5 (PMC12424212; doi:10.1186/s12912-025-03730-5)
Supplement: Supplementary file 3 — Supplementary Material 3 [file 12912_2025_3730_MOESM3_ESM.pdf]

Additional file 3 - Table school nursing theories, models/frameworks - key elements

First Author<br>Year	Name	Description/Key Points
**Theory**		
Garmy, P. 2021 [1]	School Nursing Framed by the Holistic Nursing Theory	Barbara Dossey's holistic nursing theory, holistic nursing values of integrality and awareness of whole-people and whole-system interconnectedness, nurses professional and social competences, all five theory components can be applied in several areas of school nursing. Holistic nursing is understood as „founded on the values of integrality and awareness of whole-people and whole-system interconnectedness." (Garmy et al., 2021, S. 217)<br>1. Healing<br>2. Metaparadigm of nursing theory<br>a. Nurse<br>b. Person(s)<br>c. Health<br>d. Environment (society)<br>3. Patterns of knowing<br>a. Personal<br>b Empirical<br>c. Socio-political<br>d. Ethics<br>e. Aesthetics<br>f. Not-knowing<br>4. The four quadrants<br>a. Internal individual factors—feelings and experiences b. External individual factors—behaviour and physical symptoms<br>c. Internal collective factors—group culture and values d. External collective factors—organization and system<br>5. Conclusion of all components
Broussard, L. 2007 [2]	Making a Difference: The Role of the School Nurse in the Health of Children in Schools	School Nurses' Role, situation-specific theory: Making A Difference: The School Nurse's Role in the Health of Children in the School Setting; empowerment process in the practice of school nurses, four theoretical constructs: (a) enlisting support, (b) getting through the day, (c) maintaining control over practice, and (d) adjusting to challenges.
**Role- and practice models/frameworks**		
Lewallen, T. 2015; Galemore, C. 2016;, Driscoll, L. 2021, [3–5]	Whole School, Whole Child, Whole Community (WSCC)	Model background:<br>Whole School, Whole Community, Whole Child (WSCC) build on the Whole Child approach and the coordinated school health (CSH) approach;<br>It's a Holistic Health and Education Model, each segment and layer of the WSCC model is interdependent on the others; Directly addresses the relationship between education and health.

		Whole School, Whole Community, Whole Child (WSCC) model is a unified model that supports a systematic, integrated, and collaborative approach to health and learning, supported by school environment. It is also an approach for decision making and action for health- and learning sector;<br>The student stays in the center as focal point: safe, engaged, supported, challenged, healthy;<br>Around the center is a white band which „illustrates the collaboration needed among school, health, and community sectors to implement the model" (Galemore et al., 2016, P.218).<br>Surrounded by coordination among policy, process, and practice improving learning and health; Health Education, Physical Education and -Activity, Nutrition Environment and Services, Health Services, Counselling & Psychological & Social Services, Social and Emotional Climate, Physical Environment, Employee Wellness, Family Engagement, Community Involvement; School Nurses integrated in the model (e.g. Health Education, Health Service)<br>It shows how the community and the school coordinated the needs of students ➢„school nurses recognize each student as a whole person whose health, education, social, and emotional needs must be met to obtain optimal health" (Discroll, 2021, P.194). There is an interdisciplinary team, with principals, school counselors, school nurses, behavioural health representative, family outreach counselor, community health worker from the hospital. They meet regularly to talk about the students (with special needs). Topics are e.g. student's special needs, sugar in school lunch or dental health.<br>Model Description:<br>10 components of the model: health education; physical education and activity; nutrition environment and services; health services; counselling + psychological + social services; social and emotional climate; physical environment; employee wellness; family engagement and community involvement. The last and final band is the community and shows that there have to be a collaboration between school and community.
Joint Consortium for School Health 2008, 2016; Buduhan V.H. 2021 [6, 7]	Comprehensive School Health Framework (CSH)	Comprehensive School Health (CSH) in Canada is a planned, integrated, holistic framework, student in the middle, whole school environment with actions addressing four distinct, inter-related pillars:<br>• social and physical environment,<br>• teaching and learning,<br>• healthy school policy and<br>• partnerships and services.<br>➢They are working in tandem with the school community and framework is used to guide decisions about funding and health policy; School Nursing is integrated in the role of the Public Health Nurse (PHN).<br>Provided either at school or off-site to primary and secondary school students by licensed health professionals (i.e., regulated health professionals including but not limited to nurses, psychologists, primary care physicians, audiologists, speech-language therapists, dental hygienists, dietitians, physiotherapists). Examples of common school health services are vaccination programs, mental health support services, speech-language support services, and dental screening;<br>Qualification: PHN has a baccalaureate degree in nursing and is a member of a professional regulatory body for registered nurses.
Ferro M.C. 2020 [8]	Model of professional practice of school nursing for Colombia education and health: a care bond	Model of humanistic orientation, student priority focus of action. The model is split into four meta-paradigmatic concepts, and is generating a caring bond between education and health:<br>Concept of person: students (children or adolescents) are the main subject and are taken into account with their community; also teaching staff, administrative and general personnel are subject of school nursing care;

		Concept of health: the school nurse wants to empower students to deal with their health independently, in variety ways and topics.<br>Concept of environment: this concept includes the context of the care, e.g. the classrooms, the cafeteria and recreational spaces, information management, guarantee privacy<br>Concept of nursing; the role of nursing for the school nurse: the school nurses have different roles - caring (evidence and guiding), Consulting, acute or emergency care, advocate, lead healthy, managerial, collaborators, builders and evaluators of policies, educators (health promotion), investigate update
NHS Wales 2017 [9]	A Framework for a School Nursing Service for Wales'	The framework is based on 3 levels of school nurse services:<br>Universal: provide and addresses the public health components, early identification/assessments of needs, screening, immunisations, safeguarding - for all children and young people,<br>Enhanced: identification/assessment of additional needs, offer local specialists, interprofessional cooperation (specialists and public health teams), promote health<br>Intensive: interprofessional cooperation, special needs<br>Includes Wales Safeguarding Standards for NHS School Nursing Services (safeguarding or immunisation)<br>focus: pupils and their families; Staff: minimum of one registered school nurse for each secondary school and its cluster of primary schools, leadership: service lead for the school health team; team around the child approach; different school types (primary, secondary; special schools=separate report and action plan); collaboration with public health services topics: Physical health, Mental and emotional health, Immunisation, Safeguarding, Screening;
Maughan, E. 2015, 2016; National Association of School Nurses (NASN) 2018, 2020 [10–14]	Framework for 21st Century School Nursing Practice™	The framework is based on the Whole School, Whole Community, Whole Child model. It's an Instrument to fit within this model. The framework supports the school nurse in maintaining an overview of their tasks and providing structure. The Article of 2016 includes a description how to use the 21st Framework in School Nurses' practice; many schools are already using much of the Framework in practice. The framework is intended to apply for each unique school community.<br>The focus is on the student, surrounded by the family and the community. Five different principles are built around the center, which are interrelated and cannot be prioritized. The tasks that can be assigned to the principles can therefore be assigned to two principles.<br>The five principles with examples:<br>Care Coordination: assessment of the student and family needs, case management, chronic disease management, collaborative communication, Interdisciplinary teams, Motivational interviewing/ counselling, nursing delegation, student care plans, student-centered care (student self-empowerment)<br>Leadership: Advocacy (for changes), health care and education reform, emerging models of practice, lifelong learners, technology, system level leader, professionalism<br>Quality Improvement: data collection, evaluation, research,<br>Community/Public Health: access to car, health promotion, -education, -equity, disease prevention, screening, surveillance, social determinants of health;<br>Standards of Practice: surrounded the other principles, foundational for evidence-based, Clinical Competence, -Guidelines, Code of Ethics, Critical Thinking, Evidence-based<br>Practice, Nurse Practice Acts, Scope and Standards.<br>Each principle is defined by practice components

		focus: student-centered nursing; holistic view: The framework can be a helpful tool for individuals within and outside the profession to visualize evidence-based school nursing practice in a holistic way.
Brooks, F. 2007 [15]	The school nurse as navigator of the school health journey	Public health roles of school nurses are: Leadership; Liaison and multidisciplinary partnerships, schools, services, PHC and communities; Advisory role: Sex and relationships, Personal, Health and Social Education PHSE, Promoting positive parenting; (the mentioned roles are for Health Promotion); Support and Counselling; Medical Needs; (the last two sections are for Health improvement).<br>Model Nurse as Navigator role, a leadership role; In the context of school-based public health/health promotion components the navigator concept could function as a theoretical underpinning for school nursing: The idea includes the following four components: Co-ordination of care; Information; Decision making; Self-care.
Hilsinger, G. 2006 [16]	Broad Scope of School Nursing Practice	The concept/model shows a triangle. From bottom to top there are many different interventions of school nurses. Beginning with immunisations and screenings to mild asthma and overweight in the middle up to emergency care plans at the top of the concept. Supportive learning environments, primary prevention, secondary prevention, and tertiary prevention & care. Universal and targeted activities, directed to all students to create a supportive learning environment for every student and concentrated in the few students with identified problems. Strong connection to public health model.
Wicklander, M.K. 2004 [17]	National Healthy School Standard framework (NHSS)	Strategic aims of the NHSS are:<br>- to reduce health inequalities,<br>- to promote social inclusion and<br>- to raise educational standards.<br>School nurse Role in NHSS guiding framework for the Healthy Schools Program (HSP) = public health role: Cooperation; Linking health and education partnership; partnerships; management; and working with schools; provide confidential advice and support to young people regarding health management issues due to their daily access at school; school nurses have the clinical expertise and knowledge to support school staff in the whole-school approach; assessing the health needs of the school population; link schools with primary care services.<br>Key goal of the NHSS is to have access to a school nurse (by March 2006).<br>Described barriers and support for NHSS-school nursing practice:<br>barriers: School nurses invest a significant amount of time delivering the government's immunization programs; the local partnership's limited understanding of the potential of the NHSS and the role of school nurses in bridging the health and education agendas. Reluctance on behalf of the education sector to involve school nurses and limited appreciation of the benefits school nurses bring to support efforts to raise educational attainment; lack of investment in school nurse training and professional development; varied skills, capacity, and workloads of school nursing services across the U.K -> Thus, although school nurses exist in many schools, they may not be well equipped to make contributions to local HSPs.<br>support: history of positive relationships between school nursing services and HSPs; accessible and adequate funding to local health and education partnerships provides support and mentorship for school nurses; Supportive leadership, available resources, and access to continuing education courses.
Barnfather, J. S. 1991 [18]	Modeling and role-modeling - Restructuring the role of school nurse	Using theory-driven nursing process for health promotion. School nurses' role included helping students to identify stressors so that an adequate (equilibrium or arousal) adaptive potential status was possible rather than the inadequate (impoverished) status.

		Modeling: Assessment of basic needs, psychological status, adaptive potential of the students; the school nurse determines how the student sees him/herself; basic needs and psychological attributes depending on individual internal and external resources (internal: cognitive development, spiritual/genetic factors; external: social support - family, friends, teachers and communication).<br>Role-modeling: the interventions of the school nurse adapted to the individual needs/perspective, „Nursing interventions are specifically aimed at five areas: (1) building trust, (2) affirming and strengthening resources, (3) promoting control, (4) promoting a positive orientation (including self-esteem), and (5) assisting individuals to set mutual goals directed at growth and health“ (P. 236).
Rustia, J. 1982 [19]	Rustia School Health Promotion Model	School health program which integrates the health-related functions existing within the education, service and environmental maintenance components of a school system. Nurses as the health service providers responsible for implementing the program nursing practice in the school health area would go beyond performing traditional physical health care activities (such as hearing and vision screening, first aid and immunization administration and record keeping). The unmet health care needs of today’s children and youth, (such as substance abuse, pregnancy, learning disabilities, venereal disease and behaviour disorders) make it essential to address the “total” person in health care interventions. The delineation of actual interventions in the model formally establishes the nurse’s role as encompassing a broad array of functions reflecting care of the total person. Nursing as the discipline responsible for administratoring and implementing health program activities.<br>Knowledgeable in the health and developmental needs of children and youth, and would need skills in health needs assessment, communication and interpersonal relationships, program planning and evaluation, administration, including policy formulation, systematic investigation, in the methods of utilizing health services and resources within the community. The nurse administrator would be responsible for coordinating team activities at school. Activities of the school health program will involve promoting health for students, their families, teachers, supportive personnel and the community.
Wold, S.J. 1979 [20]	School Nursing - A Framework for Practice	Framework with 5 concepts around the key point „school nursing“:<br>Public Health: School Nursing as Part of community nursing; primary, secondary and tertiary prevention; planning, implementation and follow up of activities;<br>primary: “health promotion’’ and ‘‘specific protection” activities (e.g. immunization programs)<br>secondary: conducts various screening programs (e.g. vision, hearing, and scoliosis, planning, implementation, and follow-up activities)<br>tertiary: prevention level, the school nurse works to facilitate the positive adaptation of a handicapped child in the school setting (e.g. coordination of care in school setting, counsels the child, his peers, and school personnel to enhance his acceptance in the school environment; advises school personnel regarding necessary modifications of the physical plant; home visits)<br>Adaptation: support for students on internal or external environmental change;<br>Helping Relationships: establish and maintain helping relationship<br>Tools: Nurses use tools to assess clients’ needs, adaptation, and level of wellness;<br>Systematic Process: methodical series of actions or operations employed for the achievement of specific results.
**Organisation- and delivery models/frameworks**		
Wheeler, B.A. 2017, 2019 [21, 22]	Berkshire school nursing team’s innovative service model	Service delivery model focused on the practices of the qualified school nurses - specialist community public health nurses (SCPHNs); school health based on healthy child programme Department of Health

		focus divided in two areas:<br>Safeguarding team<br>Public health team<br>Each member of the team maintain all areas of competency, which they already held and to all develop new competency around the delivery of health promotion flexibility, support colleagues and ensure priority service provision. Enable all school nurses to maintain all aspects of school nursing competency. Enable the team to meet requirements from commissioners regarding the new service specification, including safeguarding commitments. Provide regular dedicated time in schools for public health nurses, increasing the visibility and accessibility of the school nursing service for our school-aged population.<br>2019: school nurse self-reflection and evaluation process of the new model (Wheeler, 2017).
Becker S.I. 2017 [23]	Several school health delivery models	School health delivery models- Variation in Employment and Funding Sources (educational- or noneducational system). Funding: educational system (most), public health, health-care systems, other (government agencies); Employer: local hospitals, public health departments, and government agencies, related school nurses' collaboration with them; funders: local public health departments, local hospitals, most multiple/combined funding sources, partly education dollars; Focus of SN differs, all over when present/responsible for one school: day-to-day care of routine student treatments and medication needs, case management, screenings, and health education. community work to identify families needs; nurses education: registered nurses, most master qualified. 10 of 11 provided services to underserved lower socioeconomic populations. Shifting staff to more nurses resulted in provide services that included day-to-day care of routine student treatments and medication needs, case management, screenings, and health education. Models of funding and staffing models. In all interviews, the emerging models were developed to increase and expand existing services.<br>The models are not named or defined in detail. Related tasks Possible models are:<br>Registered nurse (RN): focus on tasks only the RN can do. Sometimes together with a licensed practical nurse<br>Unlicensed assistive personnel (UAP): Delegation to UAP, the RN can focus on other tasks only they can do.<br>Delivery model, information about funding and employment options.
DH CNO Professional Leadership Team 2012 [24]	A service model for school nursing	It’s a model with four stages of support for children or young people and shows the services of the school nurses. On the left side, there is a „Safeguarding“ level which covers all levels.<br>The first step is called „community“: contributing to health needs assessment, designing services to reach young people, providing services in community environments, interprofessional cooperation.<br>The next step is called „universal services“: lead, coordinate, provide services, services set out in the Healthy Child Programme, interprofessional cooperation.<br>The next step is called „universal plus“: extra help for children and families they need with „care packages“, providing care, if necessary referral or signposting to other services.<br>The last step is called „universal partnership plus“: additional services for vulnerable children or others (Long-term support), protection, safeguarding concerns.<br>Vision and service model; school nurses central to the delivery of public health agenda; school nursing understanding as universal service -focus on the student (and community for assessments);<br>qualification: qualified nurses or midwives with specialist graduate level education in community health and the health needs of school aged children and young people. Work in different settings - school related.

Sheetz, A.H. 2003 [25]	Enhanced School Health Service ESHS Model	Four core functions of local school health service:<br>(a) developing the administrative infrastructure to support high-quality services for children; (b) promoting health education, including tobacco control activities; (c) linking school health services with health care providers and public health insurance programs; and (d) implementing management information systems<br>The school health service program …<br>-…should be community based and should address the health service needs of all children in a given city or town.<br>-…should be formally established and managed by a designated BSN- or MSN-prepared school nursing leader.<br>- (the schools) should recruit BSN- or MSN-prepared professional nurses to care for students and families on the ‘‘front line.’’ (to address the complexity and diversity of student health needs)<br>-…should coordinate their efforts with all aspects of the comprehensive school health education program, as well as local community services.<br>Requirements for the school health service infrastructure:<br>Address health education and health service issues, representative committee, school nursing leader (with a baccalaureate or master’s degree in nursing, as well as community health, school health, or pediatric experience), position descriptions, a student health needs assessment, health service policies and procedures, emergency care planning, medication administration policies, and individualized health care plans for all children with special health care needs.<br>Required to provide the following for the recipient school districts: (a) monthly networking meetings for nursing leaders from recipient schools, (b) consultation on the four ESHS components, (c) site visits to the recipient districts, and (d) telephone consultation.<br>School Nurse manage programs, interprofessional team<br>Qualification: BSN- or MSN, community health, school health, or pediatric experience<br>Funding by the health department, using the state tobacco tax, Tobacco Settlement Fund;<br>Focus: student, community based;<br>Nursing staff: One full-time equivalent (FTE) certified nurse in each building with 250 to 500 students. For larger buildings, 0.1 FTE for each additional 50 students. For buildings with less than 250 students, 0.1 FTE for each 25 students.
Wisconsin State Legislative Council 1994 [26]	School Health Service Delivery in Wisconsin	School nurses employed by school districts, other agencies such as local health departments to provide school nursing services and school-based clinics. They described 6 different delivery models. Here we describe only the first one (compare Staff Brief, 1994, P.23f.) focus on school nursing, others are about school linked clinics<br>1. School-based screening, referral and follow-up services: 1. A regular nurse is hired and takes over activities such as: Emergency care, visual and hearing examinations, health examinations, vaccinations;<br>2. School-based health clinics medical services or also include social services and mental health services.<br>Qualification: registered nurse, licensed, certified by the DPI as being qualified to perform professional nursing services in a public school;<br>Rule requirements relating to the provision of school health services, e.g. standards for: material resources, staffing, staff presence, data management;<br>Focus: direct service students and staff; individual and group-level, collaboration: students, parents, professionals and community.

**Qualification models/frameworks**		
Shin, E.M. 2020 [27]	School nurse competency framework for continuing education	School nurses in Korea are obliged to complete job training as "a teacher" and continuing education as "a registered nurse"<br>Continuing education Framework addresses six core competencies:<br>Communication and collaboration with students, teaching staff, and community resources<br>School Health service implementation/programmes<br>Health education conducting<br>Legal and ethical nursing practice<br>Critical Thinking for evidence-based practice<br>Patient centred care through the integration of knowledge and skills
Keller T. 2004 [28]	Differentiated Practice Model for School Nursing	Method for standardizing, strengthening, and stabilizing school nursing within the complex school health services environment differentiation by three education levels:<br>1st Generalist (BSN),<br>2nd Master's Prepared School Nurse and<br>3rd School Nurse Analyst (PhD).<br>The dynamic of this model is dependent on close collaboration between all levels to ensure effective policies and practices.<br>Practice focus of the generalist BSN: the student, family, and school community; health assessment and education, counseling, screening, case management, and accident/acute illness services on-site and within their school community; collaborate with education colleagues in the school, community providers, and other nursing professionals in the school nurse administrative structure.<br>Practice focus of master SN: wider community, district, or region, depending their characteristics, middle management layer that relieves the BSN of broader responsibilities for the implementation and coordination of school nursing research, policies, and administration. coordination of multisite services, including supervising the implementation, administration, research, and evaluation of nursing services for the entire district (Igoe, 2000). Collaborate and interact with district school health professionals and community service providers, negotiating with these groups on behalf of school nurses, enhancing interdisciplinary communication and cooperation; some communities lack access to adequate and appropriate medical services in this case, clinical nurse specialists (CNS) and nurse practitioners (NP) offer an expanded range of health services to students.<br>Practice focus doctorally qualified SN: located at state offices (responsible for school health services), planning, implementation, and evaluation of state and federal school health policies, including data collection, data analysis, policy development, and outcomes research. responsible for assuring that school-nursing services were congruent across districts and regions, evidence-based, and consistent with professional nursing standards and with the policies developed by oversight authorities; management of conflict that arises when multiple oversight authorities formulate policies that complicate decision-making for school nurses at the local and district levels. requirement for doctoral preparation is based on a focus on the policy, rather than administrative, aspects of school health services. The skills needed for this level require extensive education in research, health sciences, and public policy, plus education credentials on par with those held by high-level school and public health officials.

References

1. Garmy P, Clausson EK, Janlöv A-C, Einberg E-L. A Philosophical Review of School Nursing Framed by the Holistic Nursing Theory of Barbara Dossey. J Holist Nurs. 2021;39:216–24. doi:10.1177/08980101211006615.
2. Broussard L. Empowerment in school nursing practice: a grounded theory approach. J SCH NURS (ALLEN PRESS). 2007;23:322–8. doi:10.1177/10598405070230060401.
3. Driscoll L. Addressing the Whole Child: The Power of Data and Teams. NASN School Nurse. 2021;36:194–6. doi:10.1177/1942602X211017533.
4. Galemore CA, Bowlen B, Combe LG, Ondeck L, Porter J. Whole School, Whole Community, Whole Child-Calling School Nurses to Action. NASN School Nurse. 2016;31:216–23. doi:10.1177/1942602X16651131.
5. Lewallen TC, Hunt H, Potts-Datema W, Zaza S, Giles W. The Whole School, Whole Community, Whole Child model: a new approach for improving educational attainment and healthy development for students. Journal of School Health. 2015;85:729–39. doi:10.1111/josh.12310.
6. Joint Consortium for School Health. What is Comprehensive School Health?: Introduction to Comprehensive School Health Promotion/Health Promoting Schools;; 2008; 2016.
7. Buduhan VH, Woodgate RL. Public health nurses in Canadian schools: An opportunity to build capacity and nursing scholarship. Public health nursing (Boston, Mass.). 2021;38:637–44. doi:10.1111/phn.12891.
8. Ferro MC, Fuentes A, Chinchilla TC, Sanchez B. Education and Health: A Care Bond. School-Nursing Model for Colombia. Investigacion y educacion en enfermeria 2020. doi:10.17533/udea.iee.v38n2e05.
9. NHS Wales. A School Nursing Framework for Wales; 2017.
10. Maughan ED, Bobo N, Butler S, Schantz S, Schoessler S, National Association of School Nurses. Framework for 21st Century School Nursing Practice: An Overview. NASN School Nurse. 2015;30:218–31. doi:10.1177/1942602X15589559.
11. Maughan ED, Duff C, Wright J. Using the Framework for 21st-Century School Nursing Practice in Daily Practice. NASN School Nurse. 2016;31:278–81. doi:10.1177/1942602X16661558.
12. Maughan ED, Bobo N., Butler S., Schantz S., National Association of School Nurses. Framework for 21st Century School Nursing Practice: National Association of School Nurses. NASN School Nurse. 2016;37(1_suppl):1S-2S PMID: 34974776 [https://www.ncbi.nlm.nih.gov/pubmed/34974776]:45–53. doi:10.1177/1942602X15618644.
13. National Association of School Nurses. Shaping Your Practice and Changing Mindsets: Framework for 21st Century School Nursing Practice TM. NASN School Nurse. 2018;33:236–8. doi:10.1177/1942602X18778292.
14. National Association of School Nurses. Framework for 21st Century School Nursing Practice™: Clarifications and Updated Definitions. NASN School Nurse. 2020;35:225–33. doi:10.1177/1942602X20928372.
15. Brooks F, Kendall S, Bunn F, Bindler R, Bruya M. The school nurse as navigator of the school health journey: Developing the theory and evidence for policy. Primary Health Care Research and Development. 2007;8:226–34. doi:10.1017/S1463423607000278.
16. Hilsinger G. Public health models, school nursing models and school administrators perceptions. NASN Newsletter. 2006;21:19–20. doi:10.1177/104747570602100112.

17. Wicklander MK. The United Kingdom National Healthy School Standard: a framework for strengthening the school nurse role. J SCH NURS (ALLEN PRESS). 2005;21:132–8. doi:10.1177/10598405050210030201.
18. Barnfather JS. Restructuring the role of school nurse in health promotion. Public health nursing (Boston, Mass.). 1991;8:234–8. doi:10.1111/j.1525-1446.1991.tb00662.x.
19. Rustia J. Rustia School Health Promotion Model. J Sch Health. 1982;52:108–15. doi:10.1111/j.1746-1561.1982.tb03963.x.
20. Wold SJ, Dagg NV. School Nursing-A Framework for Practice. Bureau Memorandum. 1979;20:2–5.
21. Wheeler BA. A new way forward: A look at one school nursing team's innovative service model. BR J SCH NURS. 2017;13:452–4. doi:10.12968/bjsn.2017.12.9.452.
22. Wheeler BA. Back to our roots: A revival of a public health nursing service. BR J SCH NURS. 2019;14:23–7. doi:10.12968/bjsn.2019.14.1.23.
23. Becker SI, Maughan ED. A Descriptive Study of Differing School Health Delivery Models. The Journal of School Nursing. 2017;33:415–25. doi:10.1177/1059840517725788.
24. DH CNO Professional Leadership Team. Getting it right for children, young people and families: Maximising the contribution of the school nursing team: Vision and Call to Action. Leeds; 2012.
25. Sheetz AH. Developing School Health Services in Massachusetts: A Public Health Model. J SCH NURS (ALLEN PRESS). 2003;19:204–11. doi:10.1177/10598405030190040401.
26. Wisconsin State Legislative Council. School Health Services in Wisconsin. Staff Brief 94-7. Madison; 1994.
27. Shin EM, Roh YS. A School Nurse Competency Framework for Continuing Education. Healthcare (Basel) 2020. doi:10.3390/healthcare8030246.
28. Keller T., Ryberg J.W. A differentiated practice model for school nursing. J SCH NURS (ALLEN PRESS). 2004;20:249–56. doi:10.1177/10598405040200050201.
